# Sheep as a Model for Liver Transplantation

**DOI:** 10.7759/cureus.42002

**Published:** 2023-07-17

**Authors:** Mohammed Alsebayel, Yasser M El-Sheikh, Falah H Al-Mohanna, Saleh I al-Abbad, Ahmed Al-Jammali, Yazeed M Alsebayel, Hamad M Al-Bahli

**Affiliations:** 1 General Surgery, Alhabib Medical Group, Riyadh, SAU; 2 Liver Transplantation and Hepatobiliary-Pancreatic Surgery, King Faisal Specialist Hospital and Research Centre, Riyadh, SAU; 3 Comparative Medicine, King Faisal Specialist Hospital and Research Centre, Riyadh, SAU; 4 Perfusion Services, King Faisal Specialist Hospital and Research Centre, Riyadh, SAU; 5 General Surgery, King Faisal Specialist Hospital and Research Centre, Riyadh, SAU; 6 Organ Transplant Center, Prince Sultan Military Medical City, Riyadh, SAU

**Keywords:** liver preservaion, animal handling, experimental sheep surgery, porcine liver transplant, experimental liver transplant

## Abstract

Objective: Experimental animal liver transplantation is the initial step, before the application of the procedure on humans. Canine and swine transplantation were used to perfect the technical aspects of the procedure. Small animals such as rats were mainly utilized to study the metabolic and immunological aspects of liver transplantation. In this paper, we describe our experience with attempting liver transplantation in a sheep animal model.

Material and method: The animal model used for both donor and recipient was outbred male weanling sheep of Naimi strain (*Ovis aries, *Awassi). They weigh between 25 and 35 kg. They were put under general anesthesia. Harvested livers were kept in cold storage. Recipients underwent hepatectomy, after construction of an active portal systemic bypass using a Medtronic pump. The implantation was done with caval replacement and direct portal anastomosis. The hepatic artery with its attachments to the aortal was anastomosed directly to the recipient aorta.

Result: Twelve pairs (24 sheep) were utilized for donor and recipient surgery. Donor surgery was completed successfully in all 12 cases. Recipient surgery was not completed in three cases, when animals were lost in the implantation phase, before reperfusion mainly due to uncontrolled bleeding, resulting in hemodynamic instability. We also lost five recipients immediately after reperfusion, mainly due to post-perfusion bleeding and hemodynamic instability. Four recipients stayed alive after the implantation.

Conclusion: We demonstrated the feasibility of using sheep as an animal model for liver transplantation. We described the similarities of sheep liver to that of humans, as well as the technical difficulties. This model is suitable in situations where other well-established models are not available for cultural or religious reasons. Further refinement in the technical aspects will be needed, as well as investigation of the biochemical outcome and long-term survival.

## Introduction

Experimental liver transplantation paved the way for the application of the procedure on humans, with subsequent outstanding clinical results. Canine and swine transplantation were the main avenue in order to perfect the technical aspects of the procedure, whereas small animals such as rats were used to study metabolic and immunological aspects of liver transplant.

Historically, one of the early animal models for liver transplantation was a canine auxiliary transplant done by Welch in 1955. Experimental orthotopic liver transplantation, however, was first described by Cannon in 1956 in an article published in Transplantation Bulletin [1]. Moore and Starzl simultaneously performed whole organ liver transplantation in dogs, utilizing two important prerequisites. The first one was cold storage of the donor liver and the second was prevention of venous congestion by veno-venous bypass [2,3,4,5].

The success of this animal model allowed for more technical and immunosuppressive refinements. These two aspects were the cornerstones for the eventual application in humans. Starzl subsequently reported a series of human liver transplants [6]. In the late 60s, swine liver transplant was successfully performed by Calne [7] which became the preferred animal model for experimental liver transplantation [8].

The use of non-human primates for experimental liver transplantation was first described by Myburgh et al. and Fortner et al. independently [9,10]. Non-human primates' liver anatomy is more similar to human than it is to dogs, which is advantageous in the study of liver transplantation. Unlike the canine liver, the liver anatomy of non-human primates is similar to that of humans.

After the initial liver transplant studies in dogs and the subsequent experience in pigs, the latter became the preferred large animal model due to its similarity to the human anatomy, as well as easy accessibility. As early as the late 1960s, several techniques of liver transplantation in pigs were described by numerous groups [11,12]. More recently a group from Barcelona described a simplified technique adopted by several groups [13].

In this paper, we describe our experience with attempting liver transplant in a sheep animal model. This is particularly important in countries where the use of pigs is prohibited for religious or cultural reasons. Advantages and disadvantages over other animal models are highlighted.

## Materials and methods

The research project was conducted at the Department of Comparative Medicine of the King Faisal Research Center, Riyadh, Saudi Arabia, after obtaining the necessary ethical approval (approval ID ORA/0152/36 dated 3rd of December, 2014 for the project # 241 055), according to the Guide for the Care and Use of Laboratory Animals of the National Research Council [14].

Animals and anesthesia

This study was part of a research project entitled “Liver Transplantation After Ex-Vivo Normothermic Perfusion”. The first phase was the construction of perfusion circuit completed [15], the second phase was liver transplantation in sheep reported in this paper, and the third will be implantation of livers that were perfused ex vivo. The choice of animals and anesthesia management was previously described in detail [15].

In short, Naimi strain (*Ovis aries, *Awassi) were used. Males weighing 30-40 Kg were selected. Acclimatization was for two weeks during which they were kept and handled according to the international regulations (Guide for the Care and Use of Laboratory Animals) [14]. Their physical suitability was ascertained by physical examination and lab investigations.

To start with, the animals were sedated with xylazine (0.05-2 mg/kg, IM, Rompun®, 2%, Bayer Health Care, Monheim, Germany). The circulatory access was through the jugular vein (16-gauge 3¼ inch percutaneous jugular catheter (Mais Co., Riyadh, Saudi Arabia). For induction propofol was used (4 mg/kg IV Propofol® 1%, Fresenius Kabi, Istanbul, Turkey). Sheep were then appropriately placed, keeping the head and neck elevated and extended. Using a laryngoscope, an endotracheal tube (ET) size 8 mm internal diameter was inserted (Mais Co.), after dripping 2 ml of 20% lignocaine (Xylocaine®, Astra Zeneca, Riyadh, Saudi Arabia) into the larynx. The sheep was pre-oxygenated and administered inhaled isoﬂurane. The sheep were ventilated with appropriate respiratory parameters. For continuous monitoring of the sheep’s hemodynamics a carotid artery catheter was inserted.

Anesthesia was maintained with isoflurane and fentanyl. Warm crystalloid solution (0.9% NaCl, 20-25 mL/kg/h) was administered prior to and following the anhepatic phase, while during the anhepatic phase, a bolus of 500 ml warm colloid solution was given.

Donor surgery

**Figure 1:**
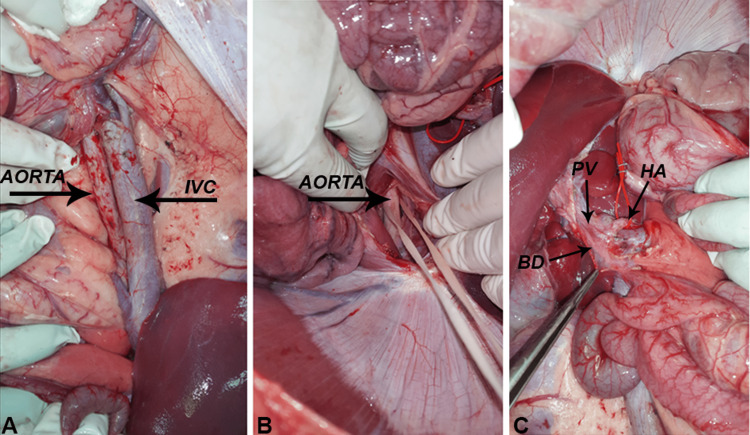
Donor Surgery "Figure reprinted from our previous paper [15].". The aorta and inferior vena cava were dissected (A). The supra-coeliac was encircled (B). The hilar structures were exposed (C). PV: Portal Vein, HA: Hepatic Artery, IVC: Inferior Vena Cava, BD: Bile Duct

The principal steps in organ retrieval are similar to that of humans and were described previously and are illustrated in Figure 1 [15]. Briefly, access to the abdomen was made through a midline incision, which was extended transversely to the right. Retracting the abdominal viscera to the left, exposed the inferior vena cava (IVC) and aorta (Figure 1A). The infra-renal aorta was then mobilized and encircled with umbilical tap.

Next was exposure of the supra-coeliac aorta which was reached by incising the diaphragmatic crura. An umbilical tape was used to encircle the aorta (Figure 1B). The hilar structures of the liver were identified and exposed (Figure 1C). Cannulation of the aorta was made to connect it to a cold saline perfusate. The lower limb circulation was excluded by ligating their vessels. To drain the circulation, the IVC was incised, and saline perfusion was continued till the drainage was clear of blood. We then perfused histidine-tryptophan-ketoglutarate (HTK, Custodiol®) preservation solution. Finally, the liver was procured. The renal and superior mesenteric vessels were ligated to free the aorta and IVC. The portal and superior mesenteric veins were dissected carefully and ligated. The hepatic artery was kept in continuity with the coeliac axis and an aortic cuff, after lighting all other celiac branches. The liver was then delivered by incising the diaphragm, IVC at the right atrium and finally the thoracic aorta.

Recipient surgery

A midline incision with bilateral sub costal extension was utilized. The exposure was difficult due to the multipartite animal’s stomach. The hilum of the liver was exposed first. The common hepatic duct was transected. The hepatic artery was dissected up to the superior aspect of the pancreas. The portal vein was dissected towards the liver and down to the pancreas. Caution was taken to avoid injury to the fragile vessels. The infra-renal aorta was dissected in preparation for future arterial anastomosis. Preparation for veno-venous bypass was made. The splenic vein was cannulated and connected to the Bio-Medicos bypass circuit, (Bio-Pump® centrifugal pump, Medtronic, Minneapolis, MN, USA), with the inflow going into the internal jugular vein.

Next was mobilization of the IVC. This vessel was almost completely enclosed by the liver except for parts of its medial and inferior aspects. The IVC was dissected carefully from the posterior abdominal wall and diaphragm. It was then encircled in its supra-hepatic. The same was done for the infra-hepatic part.

Piggyback technique was not possible in this animal model because of the complete encirclement of the IVC by liver tissue. Test clamping of the IVC was made.

The hepatic artery was then transected between ligatures. The portal vein was clamped and transected. Two clamps were put on the IVC inferiorly and superiorly and the liver was removed. This left the animal in anhepatic phase. The new liver was then brought in the field and a supra-hepatic anastomosis was performed using continuous vascular suture. The lower caval anastomosis was then completed. Next was the portal anastomosis which was done in a continuous fashion. The liver was then flushed with blood after releasing all the clamps. Hemostasis was secured. The last anastomosis was the arterial; this was done by anastomosing a cuff of the donor’s aorta attached to the coeliac access to the infra-renal aorta of the recipient. The common bile duct was cannulated with a feeding tube.

The hemodynamics of the animal was observed as well as the color and consistency of the liver. The surgical field was observed for coagulopathy and the feeding tube for evidence of bile production.

## Results

To be familiar with the anatomy of the sheep, we initially performed 10 donor surgeries, which were all completed successfully. We found donor surgery straightforward, resembling to a large extent that in humans. Bench preparation of the graft was technically simple, paying particular attention to injuries or leaks in the vascular structures. We then performed both donor and recipient procedures in a sequential manner, starting with the donor surgery.

Twelve pairs (24 sheep) were utilized for donor and recipient surgery. Donor surgery was completed successfully in all 12 cases. The recipient outcome was categorized into two groups: outcomes during implantation (three cases) and outcomes after perfusion in nine cases. 

Recipient surgery was not completed in three cases. We lost the animals in the implantation phase before reperfusion; mostly due to bleeding and hemodynamic instability. Uncontrolled bleeding occurred during the upper caval anastomosis in two cases and torsion of the fragile portal vein in one case.

Fine sutures of 5 and 6 zero polypropylene (prolene, Ethicon®, Riyadh, Saudi Arabia) were used in a continuous fashion using 4X loupe magnification. The upper caval anastomosis was challenging, especially in the left corner. Hemostatic suture could not control the bleeding. The lower caval anastomosis was also challenging, because of the difficulties encountered in exposing the anastomosis site. This was due to the large and multipartite distended stomach and bowel congestion. The wall of the lower cava however was holding the sutures well, without tearing or needle holes.

The portal anastomosis was also achievable. We were able also to construct a growth factor after completing the continuous suture. Uncontrolled bleeding was encountered once due to improper handling resulting in vessel torsion.

We also lost five recipients, immediately after reperfusion, mostly due to bleeding and hemodynamic instability. The bleeding occurred at multiple sites including the vascular anastomoses. In these five cases, it seems to be related to systemic coagulopathy likely related to reperfusion injury and/or inadequate support with blood products. In four recipients, the animals stayed alive after the implantation with hemodynamic stability. They were exterminated a few hours after the procedure since our intention was not for longer survival, but rather for technical feasibility.

The above results indicate the need for technical perfection, as well as appropriate anesthetic support, which may result in better survivals.

## Discussion

Several animal models are used worldwide for experimental surgery [6,9,10]. Swine are commonly used however their use is restricted in some countries for religious or cultural reasons [8,12]. Swine liver has five or six lobes. This anatomy does not resemble that of human liver. Because of their small size, rats are too small to be used in experimental liver transplant. A canine model use is feasible, however it is more difficult to obtain from a logistical point of view.

Though there are similarities between the swine and human liver anatomy [15], differences can create difficulties with experimental liver surgery particularly in liver resection [16].

Sheep liver shares many anatomic similarities with that of humans, such as morphology and organ-to-body weight ratio. The structures of the liver hilum (portal vein, hepatic artery) and the bile duct are similar to those of human liver. The outflow consists of three hepatic veins draining into the IVC. The left hepatic vein drains directly into the IVC. The middle hepatic vein joins the right hepatic vein instead of joining the left hepatic vein as seen in the human liver. For these anatomical and logistical reasons sheep were used in experimental liver surgery and seemed to be more convenient especially in liver resection [16-19].

The donor surgery is straightforward following the same principle in human liver retrieval. Because of the multi-parted stomach the exposure is quite difficult especially following portal clamping despite by-passing the splanchnic circulation with veno-venous bypass. The structure of the hilum is similar to humans. The portal vein is thin and fragile which requires great care with handling.

The dissection for hepatectomy is quite simple. In the sheep, the retro-hepatic IVC is mostly intrahepatic, similar to the situation in the pig model [16]. Therefore, retro-hepatic caval preservation, also known as the ‘‘piggyback’’ technique, cannot be performed in the sheep model. Hence, retro-hepatic IVC must be clamped in its supra and infra-hepatic portions and removed entirely with the liver. While the IVC is clamped, the circulating blood volume is essentially reduced in the head and neck, upper extremities, and thoracic cavity. To achieve hemodynamic stability as well as to reduce portal venous congestion, a veno-venous bypass is used to pump blood from the splenic vein to the internal jugular vein utilizing a circuit powered by a circulatory pump.

In this sheep model, the vascular anastomoses were done in a similar fashion to that in the human setting, end-to-end using a running polypropylene suture. The upper caval anastomosis can be challenging because of its thin wall. The lower caval anastomosis also can be difficult because of the distended multipartite stomach which can block the surgical field. The portal anastomosis should be done carefully because of its fragile and thin wall. Arterial anastomosis is easily done with a cuff of the aorta from the donor side.

In our experience after several attempts and passing a learning curve we manage to implant the liver successfully and kept the animal alive for a few hours after implantation with stable hemodynamics. These experiments were exploratory to assess the feasibility of liver transplant in sheep from a technical point of view. We did not attempt to specifically look at the biochemical parameters nor planned to keep the animal alive beyond a few hours after implantation.

It seems feasible to use this animal model in liver transplantation experiments, especially in countries where other established animal model is not available whether due to logistical, religious or cultural reasons.

## Conclusions

We demonstrated the feasibility of using sheep as an animal model for liver transplantation. We described the technical difficulties and the similarities to the anatomy of human liver. This model is suitable in situations where other well-established models are not available. Anatomically, sheep liver is closer to that of the human, which might be an advantage over the existing large animal models. More research is needed to refine the technical aspect.
